# Silver Nanoparticles/Ibuprofen-Loaded Poly(l-lactide) Fibrous Membrane: Anti-Infection and Anti-Adhesion Effects

**DOI:** 10.3390/ijms150814014

**Published:** 2014-08-12

**Authors:** Shuai Chen, Guangda Wang, Tianyi Wu, Xin Zhao, Shen Liu, Gang Li, Wenguo Cui, Cunyi Fan

**Affiliations:** 1 Department of Orthopaedics, Shanghai Sixth People’s Hospital, Shanghai Jiao Tong University School of Medicine, 600 Yishan Road, Shanghai 200233, China; E-Mails: hikev00@163.com (S.C.); liushensjtu@126.com (S.L.); 2 Department of Orthopaedic, Yuhuangding Hospital of Yantai, 20 Yuhuangding East Road, Yantai 264000, China; E-Mail: xlnedmd@126.com; 3 Department of Orthopaedics and Traumatology, Faculty of Medicine, the Chinese University of Hong Kong, Hong Kong, China; E-Mails: bxnmephd@126.com (T.W.); jgl100n@126.com (G.L.); 4 Orthopedic Institute, Soochow University, 708 Renmin Road, Suzhou 215006, China; E-Mail: crpi688@126.com

**Keywords:** peritendinous adhesions, antimicrobial polymers, electrospun membrane, silver nanoparticle, ibuprofen

## Abstract

Infection caused by bacteria is one of the crucial risk factors for tendon adhesion formation. Silver nanoparticles (AgNP)-loaded physical barriers were reported to be effective in anti-infection and anti-adhesion. However, high silver load may lead to kidney and liver damages. This study was designed for Ibuprofen (IBU)-loaded poly(l-lactide) (PLLA) electrospun fibrous membranes containing a low dosage of Ag to evaluate its potential in maintaining suitable anti-infection and good anti-adhesion effects. The *in vitro* drug release study showed a sustained release of Ag ions and IBU from the membrane. Inferior adherence and proliferation of fibroblasts were found on the Ag4%–IBU4%-loaded PLLA electrospun fibrous membranes in comparison with pure PLLA and 4% Ag-loaded PLLA membranes. In the antibacterial test, all Ag-loaded PLLA electrospun fibrous membranes prevented the adhesion of *Staphylococcus aureus* and *Staphylococcus epidermidis*. Taken together, these results demonstrate that Ibuprofen is effective in enhancing the anti-adhesion and anti-proliferation effects of 4% Ag-loaded PLLA fibrous membrane. The medical potential of infection reduction and adhesion prevention of Ag4%–IBU4%-loaded PLLA electrospun fibrous membrane deserves to be further studied.

## 1. Introduction

Posttraumatic tendon adhesion is the most common complication after surgical repair of tendon injury [1,2]. Adhesion that restricts normal tendon gliding may form between the tendon and the surrounding tissues and thus leads to, e.g., hand disability. According to the pathology of tendon adhesion, infection induced by bacteria is one of the crucial risk factors for adhesion formation [3,4]. Such infection reaction has very limited capacity to heal spontaneously and the tendon adhesion would therefore deteriorate if left untreated.

Recently, in order to prevent adhesion induced by infection, anti-infection and anti-adhesion Ag-loaded physical barriers have been used [5,6]. Although the prepared Ag-loaded physical barriers were defined as non-cytotoxic, high silver load may still lead to kidney and liver damages after delivery of Ag ions by the blood circulatory system [7,8,9]. The cytotoxicity of Ag would be reduced with less drug load, though the anti-infection and anti-adhesion effect would thus be inevitably weakened. Therefore, how to maintain the low-cytotoxicity while keeping satisfactory function is the key to the optimization of a clinic treatment program.

Electrospun fibrous membrane is famous for tissue separation [10,11,12], as the microporous structure allows the exchange of nutrients and, above all, prevents the immigration of fibroblasts from outside the tendon sheath to the tendon inside [13,14,15]. Ibuprofen (IBU) can be loaded into the fibers to enhance the anti-adhesion effect of physical barriers with low cytotoxicity for its chemical composition and pharmacological action [16,17]. However, the bacterial infection cannot be prevented by sole IBU. Therefore, the combination of IBU and Ag is a good choice for decreasing kidney and liver damages caused by high dose of Ag while maintaining good anti-adhesion effect. In this study, silver nanoparticles Ag and IBU are mixed and, subsequently, electrospun into the nanofibers directly. It is expected that the drug load of Ag can be reduced without significant weakening of anti-infection and anti-adhesion effects in Ag/IBU-loaded poly(l-lactide) (PLLA) membrane. The Ag/IBU-loaded electrospun fibrous membrane was characterized by Ag and IBU release study and evaluated for its ability to inhibit fibroblasts adhesion and prevent infection.

## 2. Results

### 2.1. Characterization of Electrospun Poly(l-lactide) (PLLA) Fibrous Membranes

**Figure 1:**
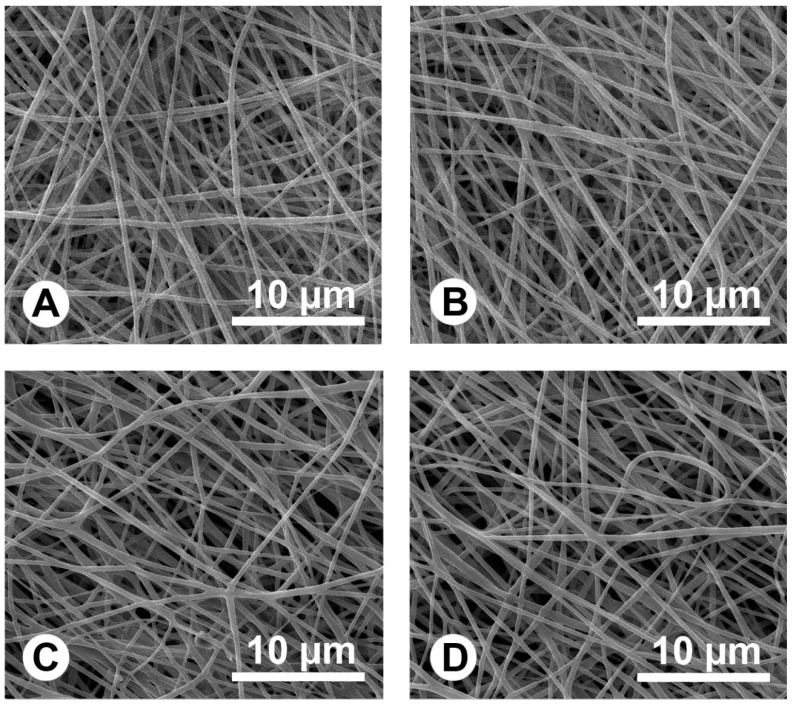
Scanning electron microscopy (SEM) images of electrospun fibers of poly(l-lactide) (PLLA) (**A**); Ag4%–PLLA (**B**); Ag4%–IBU4%–PLLA (**C**); and Ag8%–PLLA (**D**).

**Figure 2:**
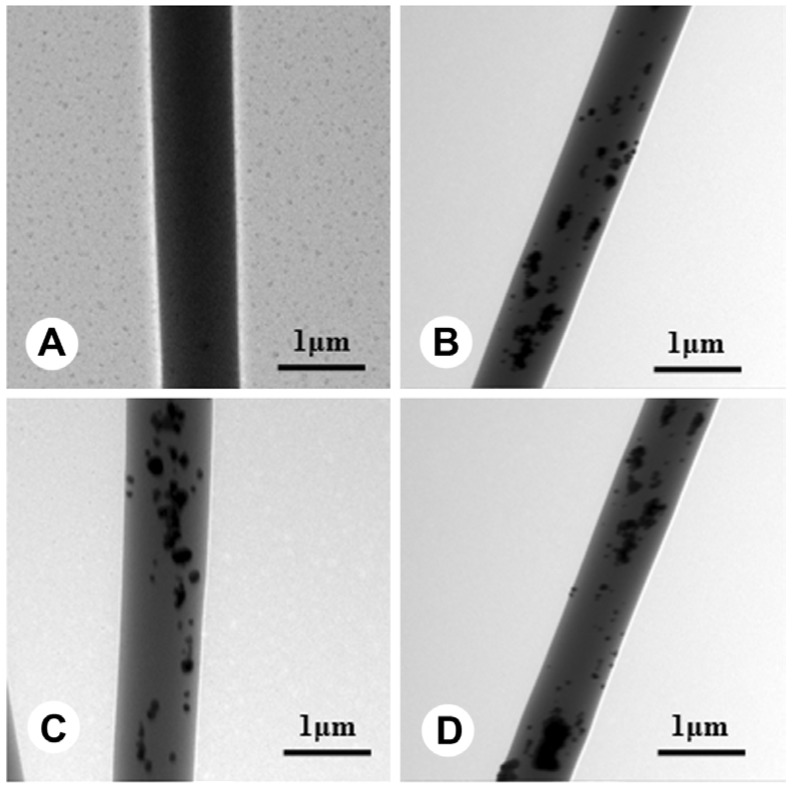
Transmission electron microscope (TEM) images of electrospun fibers of PLLA (**A**); Ag4%–PLLA (**B**); Ag4%/4% ibuprofen (IBU)–PLLA (**C**); and Ag8%–PLLA (**D**).

The thicknesses of all fibrous membranes were near 200 μm. The fibers morphology was observed using scanning electron microscopy (SEM), and it can be seen that there are no beads in the fibrous structures and the fibers are uniform in size, forming randomly interconnected structures and seemingly smooth in all samples (Figure 1). The Ag could be found inside the fibers from transmission electron microscope (TEM) images, and all Ag were successfully wrapped into fibers even though some agglomerated particles were shown in the fibers (Figure 2). Fiber diameter was 1.02 ± 0.26, 1.14 ± 0.24, 1.21 ± 0.37 and 1.18 ± 0.42 μm in PLLA, Ag4%–PLLA, Ag4%–IBU4%–PLLA and Ag8%–PLLA, respectively. To clarify the effects of the Ag and IBU on the surface properties of electrospun fibers, water contact angles of electrospun fibrous membranes were measured. The water contact angles were 131.3° ± 3.1°, 125.1° ± 4.1°, 126.8° ± 3.9°, and 118.4° ± 2.7° for PLLA, Ag4%–PLLA, Ag4%–IBU4%–PLLA and Ag8%–PLLA, respectively.

### 2.2. In Vitro Drug Release

**Figure 3:**
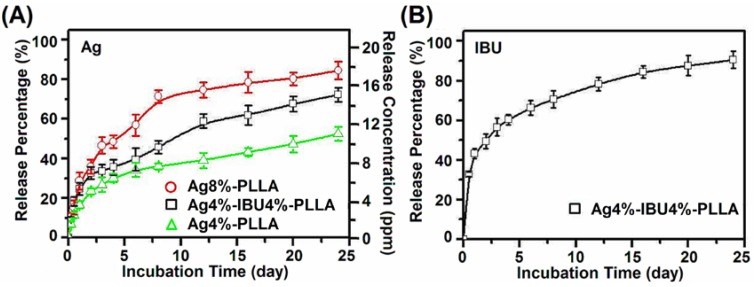
*In vitro* Ag ion cumulative release percentage and release concentration from Ag4%–PLLA, Ag4%–IBU4%–PLLA and Ag8%–PLLA electrospun fibers (**A**); and IBU release from Ag4%–IBU4%–PLLA electrospun fibers (**B**) after incubating in PBS at 37 °C.

The Ag ions release profiles of the PLLA electrospun fibrous membranes were shown on vertical axis (*y*1) of Figure 3A. During the initial 2 days, burst release of Ag ions from the medicated Ag4%–PLLA, Ag4%–IBU4%–PLLA and Ag8%–PLLA electrospun fibrous membranes were 23.5%, 32.7% and 35.9%, respectively. In the following 10 days, the samples of Ag4%–PLLA, Ag4%–IBU4%–PLLA and Ag8%–PLLA took on a sustained release phase and, especially, Ag ions were nearly completely released in Ag8%–PLLA at the last time point. *In vitro* Ag release cumulative concentration (ppm) from electrospun fibrous fibers was also summarized on the vertical axis (*y*2) of Figure 3A. The concentrations of Ag ions released during the initial 24 days were about 11.21, 13.36 and 15.89 ppm in the fibrous membranes with 4.0%, 4.0% and 8.0% AgNP entrapment for Ag4%–PLLA, Ag4%–IBU4%–PLLA and Ag8%–PLLA, respectively. The IBU release profile of Ag4%–IBU4%–PLLA electrospun fibers is shown in Figure 3B. During the first 2 days, burst release of ibuprofen from the membrane was 49.5%, followed by a sustained-release in the subsequent 10 days. In brief, drug release behavior depends primarily on polymer matrix degradation, drug diffusion and Ag release (Figure 3).

### 2.3. In Vitro Cell Adhesion

**Figure 4:**
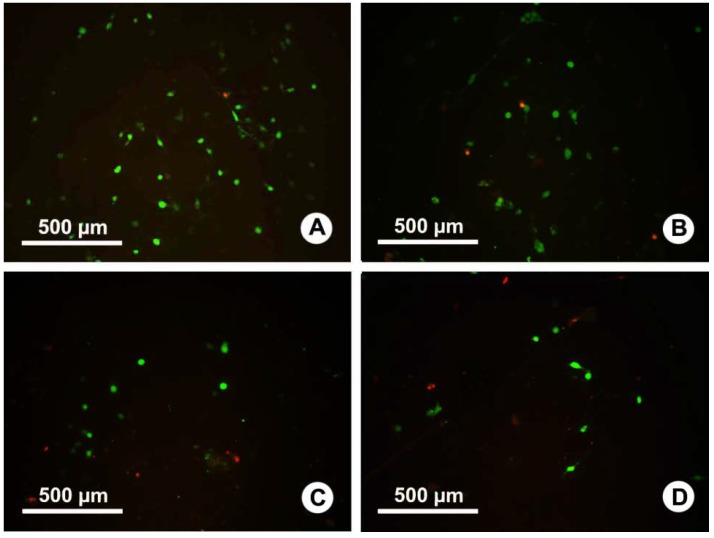
The results of a viability assay of C3H10T1/2 cells (C3) grown on the surface of PLLA (**A**); Ag4%–PLLA (**B**); Ag4%–IBU4%–PLLA (**C**); and Ag8%–PLLA (**D**). Red: dead cells; Green: live cells.

The viability of C3 cells on the surface of PLLA fibrous membranes (with or without Ag or IBU) was compared after 24 h culture (Figure 4). Our results showed that live cells can grow on the surfaces of all PLLA fibers membranes, but fewer cells were found on drug-loaded fibrous membranes. Adherence on the 8% Ag-loaded PLLA fibrous membrane was inferior to that on the 4% Ag-loaded fibrous membrane. In addition, fluorescent micrographs showed the least adherence of C3 fibroblasts on Ag/IBU-loaded PLLA fibrous membrane after 24 h incubation (Figure 4). The dead cells on different surfaces showed the opposite trend to the live cells on the respective surfaces.

### 2.4. In Vitro Cell Proliferation

**Figure 5:**
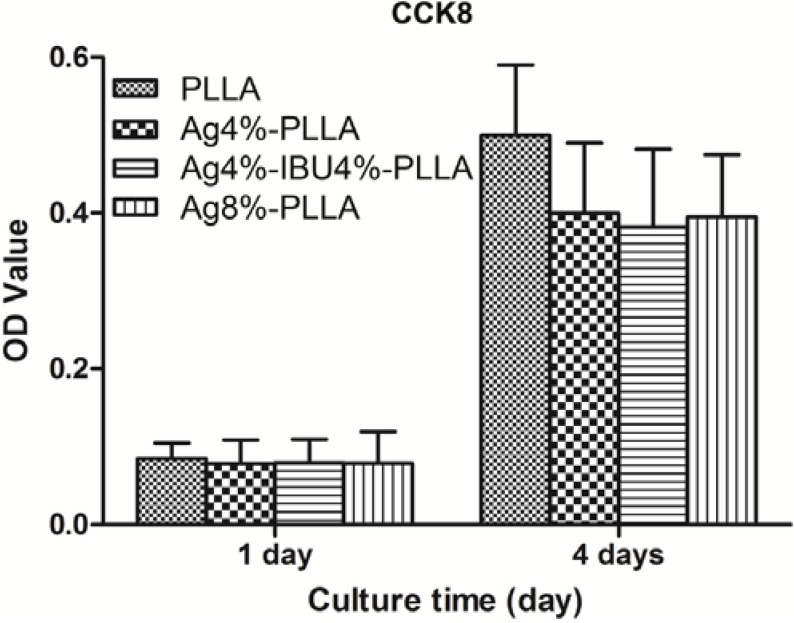
The results of a viability assay of C3H10T1/2 cells (C3) grown on the surface of PLLA, Ag4%–PLLA, Ag4%–IBU4%–PLLA and Ag8%–PLLA.

The proliferation of NIH3T3 cells on the surface of different PLLA fibrous membranes was compared after 1 and 4 days (Figure 5). It was observed that the cells grew on all kinds of surfaces as over time. However, lower viability cells were detected on the surface of Ag-loaded PLLA fibrous membrane compared with the surface of PLLA fibrous membrane. By comparing the viability of cells on different surfaces, the cells proliferated worse on the surfaces of drug-loaded PLLA fibrous membranes than un-loaded PLLA fibrous membranes after 4 days; the cell growth on the Ag/IBU-loaded fibrous membrane after 4 days showed the worst viability. However, the observed differences for drug-loaded fibrous membranes were not statistically significant.

### 2.5. Anti-Bacterial Test

**Figure 6:**
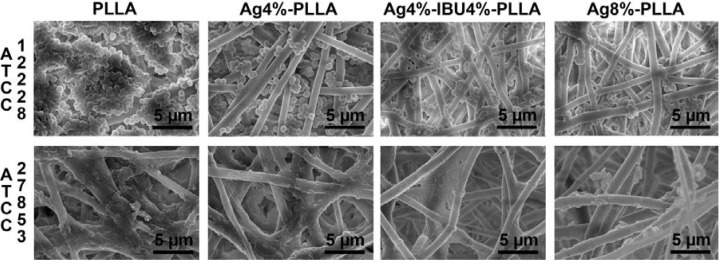
The SEM images of bacteria on the surface of different electrospun fibers after 1 day culture.

The attachments of two bacterial strains on different surfaces were investigated using SEM after 24 h culture (Figure 6). The images revealed a clear difference among the number of all bacteria on the surfaces of each sample. The SEM images showed that the bacteria on pure PLLA fibers were dense. However, the bacteria on Ag or Ag/IBU-loaded fibers were sparse, indicating a better antibacterial ability against these two stains, although there seems to be fewer bacteria on drug-loaded PLLA fibrous membrane surface with high Ag loading.

## 3. Discussion

In this study, we tested the anti-infection and anti-adhesion abilities of various Ag-loaded PLLA fibrous membranes. The electrospun fibrous membranes were prepared to prevent peritendinous adhesion and reduce infection with the expectation that the drug load of Ag can be reduced without significant impairment of anti-adhesion effect after the incorporation of IBU. The Ag-loaded electrospun fibrous membranes can release Ag (and IBU) for at least three weeks, which enables the membranes to inhibit fibroblasts adhesion and prevent bacteria attachment. The incorporation reduces the drug load of Ag without obvious reduction of the anti-adhesion effect.

Silver ions are widely used for anti-infection due to the powerful antibacterial ability [18,19]. Even if a low amount of Ag is loaded on the membrane to insure its non-cytotoxicity, according to ISO instructions (ISO, International Organization for Standardization), the potential risk of the Ag delivery in the blood circulatory system related to the decrease in kidney and liver activity cannot be ignored. In our study, the anti-bacterial effect of Ag-loaded fibrous membranes were confirmed and, moreover, after the incorporation of IBU into 4% Ag-loaded fibrous membranes, the anti-bacterial effect was maintained and not worse than the 8% Ag-loaded membrane.

Physical barrier loaded with IBU could be used to prevent cell adhesion, proliferation and further *in vivo* peritendinous adhesion. After incorporated into the electrospun membranes, IBU would enhance the anti-adhesion function of the electrospun membrane. In this study, the Ag4%/4% IBU-loaded fibrous membrane exhibited better anti-adhesion and anti-proliferation abilities than other two silver loaded fibrous membranes Therefore, IBU can still enhance the anti-adhesion effect of the 4% Ag-loaded electrospun membrane. Furthermore, damage to kidney and liver was reduced with low silver load at the same time.

Acute inflammatory response may also increase the incidence of postoperative adhesion. Although anti-inflammatory physical barriers have been used to prevent peritendinous adhesion and inflammatory response, high risk of infection caused by biomaterial application was still a daunting challenge for surgeons. IBU-loaded PLLA fibrous membrane can prevent the inflammatory reaction. Therefore, it could be expected that the anti-infection and anti-inflammation effects of the Ag/IBU-loaded PLLA fibrous membrane can be combined together. However, there are limitations in our study. Differences that were not statistically significant limited the efficiency of our analysis. Further study, especially *in vivo* experiments, should be performed to test the anti-bacterial and anti-adhesion effect before clinic use.

## 4. Materials and Methods

### 4.1. Materials

Poly(l-lactide) (PLLA, *M*_W_ = 50 kDa, *M*_W_/*M*n = 1.6) was prepared by bulk ring-opening polymerization of l-lactide using stannous chloride as an initiator (Jinan Daigang Co., Jinan, China). Silver nanoparticles (Ag, purity 99.9%, diameter, 60–120 nm) were purchased from the Aladdin Regents Company (Shanghai, China). Ibuprofen, CCK8, acridine orange, and propidium iodide were supplied by Sigma-Aldrich (Saint Louis, MO, USA). *Staphylococcus epidermidis* (ATCC12228) and *Pseudomonas aeruginosa* (ATCC27853) were purchased from Chuangxiang Biotechnology (Shanghai, China). Dulbecco’s modified Eagle’s medium (DMEM) and fetal bovine serum were purchased from Gibco (Grand Island, NY, USA). All other chemicals and solvents were of reagent grade or better and purchased from GuoYao Regents Company (Shanghai, China) and used without further purification, unless otherwise indicated.

### 4.2. Fabrication of Electrospun Fibrous Membranes

Electrospinning was carried out according to our previous study [13,17,20,21]. Electrospinning solution is fabricated using 1 g PLLA and 4 g dichloromethane (DCM) and 2 g *N*,*N*-dimethlformamide (DMF) for fabricating PLLA fibers. Zero point zero four grams Ag, 0.04 g Ag/0.04 g IBU and 0.08 g Ag were completely dispersion in 2.0 g DMF, respectively. One gram PLLA was completely dissolved in 4.0 g DCM. Then, 0.04 g Ag, 0.04 g Ag/0.04 g IBU and 0.08 g Ag solution were mixed with PLLA solution for fabricating Ag4%–PLLA, Ag4%/4% IBU–PLLA and Ag8%–PLLA fibers, respectively. The electrospinning parameters are 15 kV using a high-voltage power supplier (Tianjing High Voltage Power Supply Co., Tianjin, China), flowing rate 3.0 mL/h (syringe pump, Lange, Baoding, China), and collect distance of 15 cm. All fibers were vacuum dried at room temperature for 1 day before using.

### 4.3. Characterization of the Electrospun Fibrous Membranes

The morphology was observed by scanning electron microscopy (SEM, FEI Quanta 200, FEI Co., Eindhoven, The Netherlands). At least five images of each sample (10,000× magnification) were obtained. The mean diameter of the fibers was measured with Photoshop 8.0 using at least 20 different fibers and 200 different segments from each image selected randomly. The structure of Ag in the PLLA electrospun fibers was observed with a transmission electron microscope (TEM, JEM-2100F, JEOL, Tokyo, Japan).

Water contact angle of the fibrous membranes was tested to evaluated surface wettability at room temperature using a Krüss GmbH DSA 100 Mk 2 goniometer (Krüss GmbH, Hamburg, Germany), followed by image processing of sessile drop profiles with DSA 1.8 software (Krüss GmbH, Hamburg, Germany).

### 4.4. In Vitro Drug Release Study

For evaluating the *in vitro* release behavior of Ag ions, the Ag nanoparticles (AgNP)-loaded PLLA fibrous membranes were first punched into small squares with a total mass of *ca.* 100 mg, which were immersed in 20 mL of 154 mM phosphate buffered saline (PBS, pH 7.4), containing 0.02% sodium azide as a bacteriostatic agent. The suspension was kept in a thermostated shaking water bath (Taichang Medical Apparatus Co., Taichang, Jiangsu, China) with a shake speed of 50 cycles per minute at 37 °C. At predetermined time intervals, 5.0 mL of the release buffer was removed for analysis and 5.0 mL of fresh PBS was added back for continuing incubation.

The amount of cumulatively-released IBU molecules in the collected medium was tested by UV–vis spectroscopy at 264 nm. A standard calibration plot of IBU molecules in the concentration range of 0–0.05 mg/mL (264 nm absorbance) was used to determine the IBU concentration. The percentages of the released IBU were then calculated based on the initial weight of IBU incorporated in the electrospun scaffold. The results are presented in terms of cumulative release, which was calculated according to the cumulative amount of release equation [22].

### 4.5. Anti-Adhesion Test

#### 4.5.1. *In Vitro* Cell Culture

NIH3T3 cells were used to evaluate the adhesion and proliferation on the electrospun fibrous membrane surfaces. The cells were incubated in complete culture medium at 37 °C in a humidified atmosphere with 5% CO_2_. The medium was changed every 3 days. After 75% confluence, the NIH3T3 cells were harvested with 0.25% trypsin for cell seeding. The electrospun membranes in a 24-well plate (Costar 3548, Corning, New York, NY, USA) were sterilized by immersion in 75% ethanol for 1.5 h and then washed twice with PBS to remove residual ethanol.

#### 4.5.2. Fluorescent Staining and Observation

The presentation of NIH3T3 cells on the electrospun fibrous membranes was evaluated by fluorescent microscopy (LEICA DM 4000 B, LEICA, Wetzlar, Germany). Cells (1 × 10^5^ cells/mL) were seeded into each well (400 μL/well) and incubated for 24 h. Then, these cells on the electrospun fibrous membranes were stained with acridine orange and propidium iodide. Thirty minutes later, the cells were observed under the fluorescence microscope. After light excitation, the nuclei of living cells were stained bright green, while the dead cells were stained red.

#### 4.5.3. Cell Proliferation Assay

Cell proliferation was determined using a Cell Counting Kit (CCK-8) assay according to the manufacturer’s instruction. Cells (1 × 10^5^ cells/mL) were seeded into each well (200 μL/well) and incubated for 24 h. Twenty microliters of CCK-8 buffer was added to each well, and cells were incubated at 37 °C for an additional 4 h. The solution (200 μL) was transferred into a 96-well plate to determine the absorbance at 450 nm using a spectrophotometer (Synergy 2; BioTek, Winooski, VT, USA). The cells were washed with PBS (500 μL) and incubated for an additional 3 days with DMEM (200 μL) till another CCK-8 assay at day 4.

### 4.6. Bacterial Inhibition Test

#### 4.6.1. Bacteria Culture

*S. epidermidis* (ATCC12228) and *P. aeruginosa* (ATCC27853) were, respectively, cultured on Trypticase soy agar (TSA; BD Biosciences, Franklin Lakes, NJ, USA) medium at 37 °C overnight. Then, the single stain was cultured in 10 mL BBL Trypticase soy broth (TSB) for 12 h. After culture, each stain was adjusted to a concentration of 1 × 10^6^ CFUs/mL in TSB according to McFarland standard. Samples were cultured in 1 mL of suspension with agitation at 100 rpm.

#### 4.6.2. Scanning Electron Microscopy (SEM)

SEM was used to observe the attachment of bacteria. After 24 h culture, samples were washed three times with PBS and fixed in 2.5% glutaraldehyde for 2 h at 4 °C. Dehydration of samples was performed with increasing concentrations of ethanol. Then, the samples were freeze-dried, coated with gold and observed using a scanning electron microscope (SEM, FEI Quanta 200).

### 4.7. Statistical Analysis

Results were expressed as mean ± standard deviation. Statistical software SPSS 10.0 (SPSS, Chicago, IL, USA) was used to analyze the data by one-way analysis of variance (ANOVA); *p* < 0.05 was considered significant.

## 5. Conclusions

The electrospun Ag/IBU-loaded PLLA fibrous membrane could not only prevent cell adhesion and proliferation but also reduce bacterial infection through its stable release of silver ions and IBU. Ibuprofen was demonstrated to be a good method to enhance the anti-adhesion and anti-proliferation effects of 4% Ag-loaded PLLA fibrous membrane. The Ag4%/4% IBU-loaded PLLA electrospun fibrous membrane deserves to be further studied for its potential as a multifunctional barrier in clinical application.
